# Guide wire misadventure: a duo of inadvertent intravascular migration

**DOI:** 10.1093/jscr/rjaf431

**Published:** 2025-06-18

**Authors:** Peter O Adeoye, Somto E Nwabueze, John Awodi, Denen Atsukwei, Babajide O Ayandele

**Affiliations:** Division of Thoracic and Cardiovascular Surgery, University of Ilorin and University of Ilorin Teaching Hospital, Oke Ose, P.M.B. 1459, Ilorin, 240001, Kwara State, Nigeria; Division of Thoracic and Cardiovascular Surgery, Department of Surgery, Federal Medical Centre, Old Akwanga Road, P.M.B. 1004, Keffi, Nasarawa State, Nigeria; Division of Thoracic and Cardiovascular Surgery, University of Abuja Teaching Hospital, Gwagwalada, Abuja, Federal Capital Territory, P.M.B. 228, Abuja, Nigeria; Department of Radiology, Federal Medical Center, Old Akwanga Road, P.M.B 1004, Keffi, Nasarawa State, Nigeria; General Surgery Division, Department of Surgery, Federal Medical Center, Old Akwanga Road, P.M.B. 1004, Keffi, Nasarawa State, Nigeria

**Keywords:** central venous catheterization, Seldinger technique, guide wire migration, intrathoracic complication

## Abstract

Central venous catheterization (CVC) is an integral part of patient care in the intensive care unit. The Seldinger technique, known for its minimal invasiveness, has become the standard for CVC insertion. Despite the technique’s effectiveness and safety, guide wires—integral to the procedure—carry risks, including a rare but serious complication: intrathoracic migration of a retained guide wire. Although the overall complication rate for CVC insertion is around 11.8%, intrathoracic migration of a guide wire has not been previously reported, underscoring its rarity and the necessity of meticulous guide wire management. We present a case series of two patients who experienced this rare complication. In the first case, a 25-year-old male developed a right hemothorax after the guide wire migrated into the thoracic cavity. In the second case, a 49-year-old female had a retained intravascular guide wire that migrated from the subclavian vein to the femoral vein, complicated by thrombosis. Both patients underwent successful retrieval and treatment through a multidisciplinary approach. These cases highlight the importance of vigilant guide wire management and prompt recognition of this potentially life-threatening complication. This case series emphasizes the need for healthcare providers to be aware of the risks associated with central venous catheterization and to adopt a multidisciplinary approach to manage these rare but serious complications.

## Introduction

**Figure 1:**
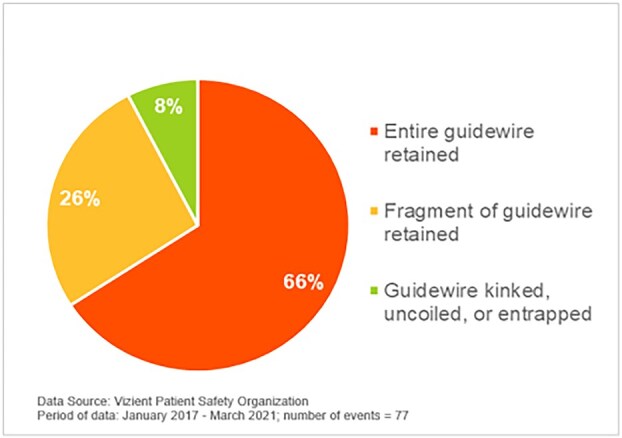
Description of guidewire issue. Source: Vizient Patient Safety Organization Safety Alert, 2022.

Central venous catheterization (CVC) is a common medical procedure used for fluid resuscitation, parenteral nutrition, hemodialysis and hemodynamic monitoring. Despite its routine use, complications associated with CVCs occur in nearly 15% of patients, with mechanical complications being the most common, occurring in 5%–19% of cases [1]. Guide wire retention, although rare, is a serious complication that can arise from factors such as operator fatigue, inexperience, or inadequate supervision. The true incidence of guide wire remains poorly defined; however Vizient® Patient Safety Organization (PSO) conducted a retrospective analysis of event reports involving retained central line guidewires to highlight their persistence, common causes and outcomes, and identify system improvements to prevent their occurrence. From January 2017 through March 2021, a search of the Vizient PSO database yielded 77 event reports involving a retained central venous or arterial line guidewire. Of those events, 51 (66%) involved an entire guidewire accidentally left in the patient, 20 (26%) involved a fragment of a wire, and 6 (8%) involved a guidewire that was entrapped in the catheter or kinked or uncoiled (near misses) during the procedure (Fig. 1) [2, 3]. We present a unique case of a 25-year-old male patient who underwent CVC placement for fluid resuscitation and parenteral nutrition, but experienced an unintended guide wire migration into the thoracic cavity, resulting in right hemothorax and a 49-year-old female who had a retained intravascular guide wire that migrated from the subclavian vein to the femoral vein, complicated by thrombosis. This report highlights the importance of vigilant guide wire management and a multidisciplinary approach to retrieval, involving interventional radiology and cardiothoracic surgery, to prevent potentially fatal outcomes.

## Case reports

### Case 1

#### Clinical history

A 32-year-old male patient was admitted to the general surgery division for management of a chronic high-output enterocutaneous fistula, a complication of an exploratory laparotomy performed three weeks prior at a peripheral hospital. The initial surgery was necessitated by a penetrating gunshot abdominal injury.

During resuscitation, central venous catheterization was performed via the right subclavian approach to facilitate fluid resuscitation and parenteral nutrition. However, control of the guide wire was inadvertently lost during catheter insertion, resulting in guide wire migration. Initial attempts to retrieve the guide wire using the suck-out technique and local exploration through a 4 cm supraclavicular incision were unsuccessful. The patient remained asymptomatic with normal vital signs and unremarkable chest examination findings for ⁓48 h post-procedure. However, he subsequently developed reduced breath sounds in the right lower lung zone, indicating a potential complication.

#### Diagnostic imaging

**Figure 2:**
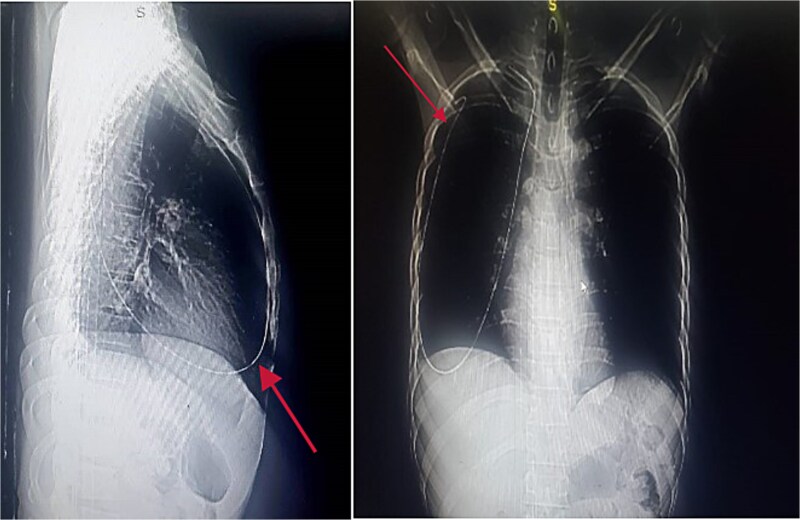
Chest X-ray posterior anterior and lateral view showing the intrathoracic metallic guide. The wire’s thin, linear shape is clearly visible, with an abrupt change in course around the cupola indicating its unintended migration from the subclavian vein into the chest cavity.

An immediate chest X-ray revealed the metallic guide wire within the right hemithorax, coursing from the anterior medial aspect of the cupola to the posterolateral aspect of the second intercostal space, with a notable curve at the diaphragm (Fig. 2).

**Figure 3:**
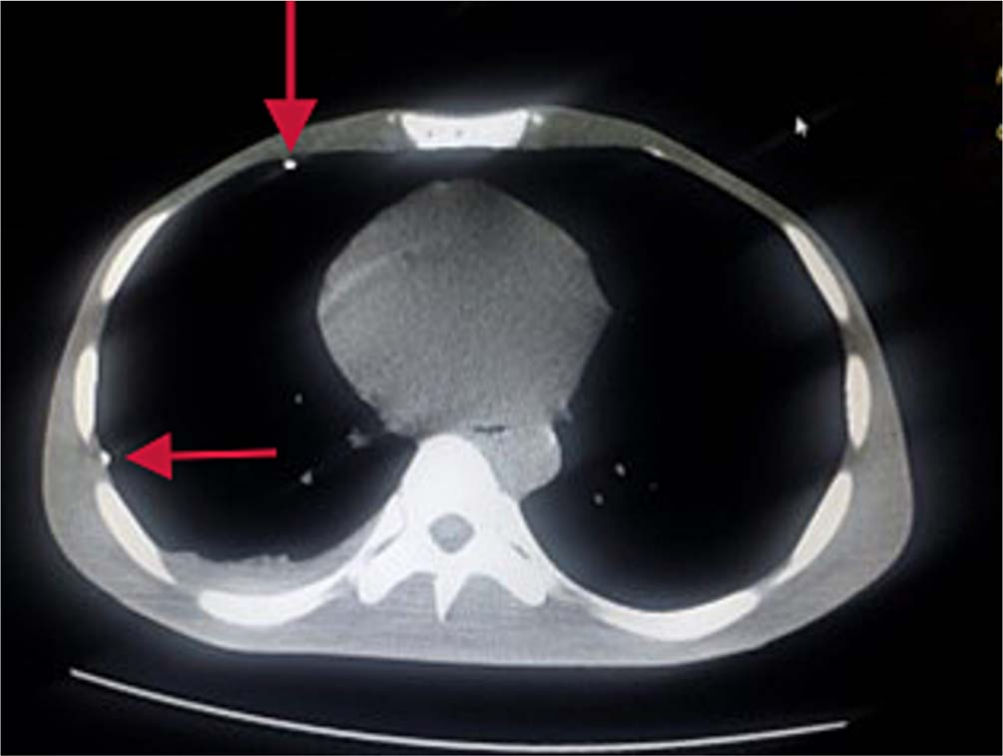
Thorax CT showing guide wire in the pleural space.

**Figure 4:**
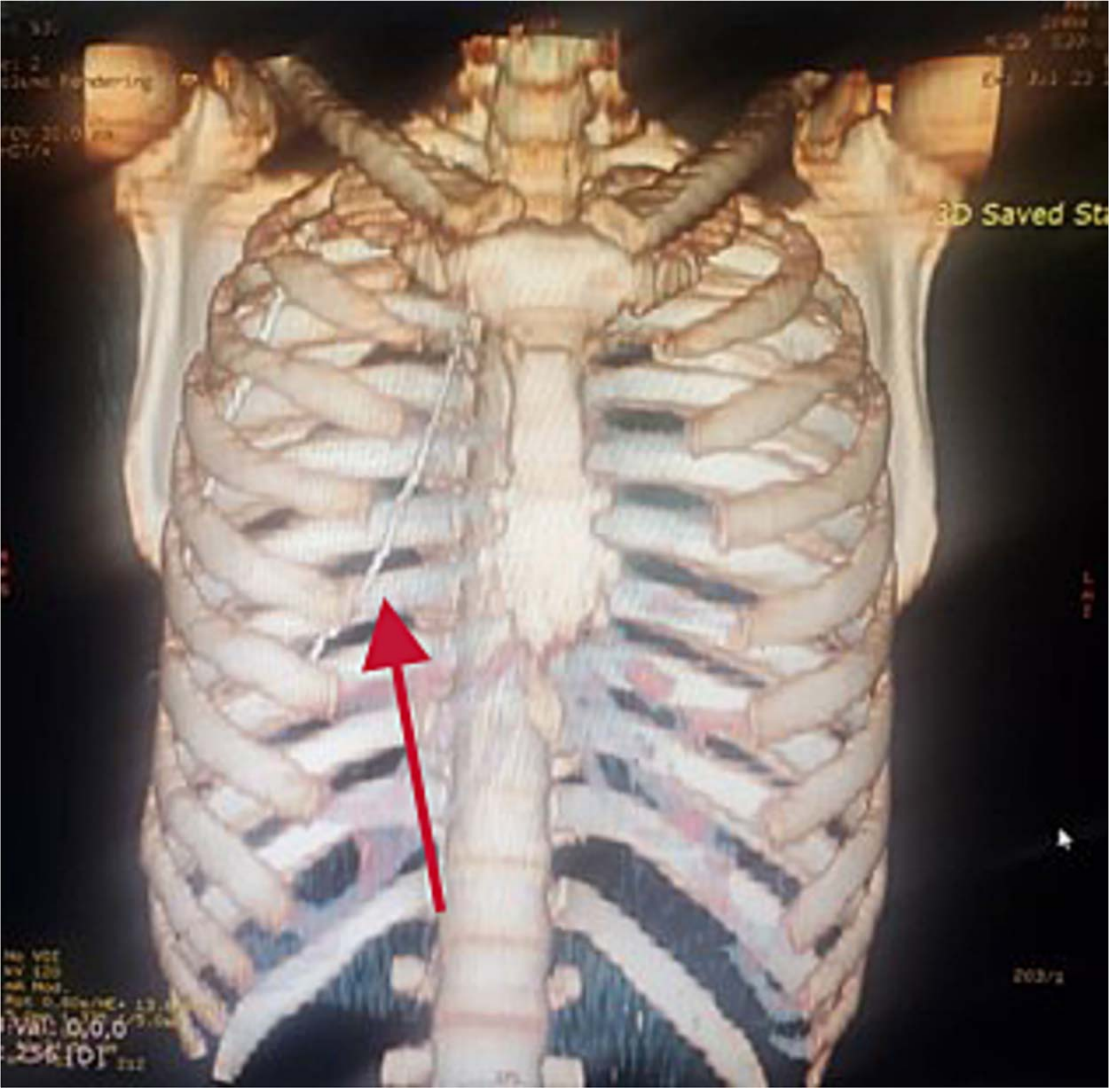
Reconstructed 3D image of the CT scan.

Further evaluation with chest ultrasound and computed tomography (CT) thorax, performed 48 h later, confirmed the guide wire’s position (Figs 3 and 4). The imaging studies demonstrated that the tail end of the guide wire was lodged in the right subclavian vein, while the remainder was situated in the pleural space of the right hemithorax. Notably, the guide wire was in contact with the 7th rib along the anterior and mid-axillary line. Additionally, a moderate right hemothorax had developed.

#### Management and surgical intervention

The patient was resuscitated and prepared for surgery via a 16G peripheral line. A mini-thoracotomy via the 7th intercostal space was performed under local anesthesia and sedation, during which the guide wire was successfully retrieved. A 28 French chest tube was placed before transitioning the patient to general anesthesia for the laparotomy.

#### Post-operative course

The post-operative period was uneventful. The patient was extubated 72 h following the procedure and showed satisfactory recovery.

### Case 2

#### Clinical history

A 49-year-old female patient was admitted for a left total hip replacement due to avascular necrosis of the left femoral neck. During preoperative workup, a central venous catheter was inserted via the right subclavian approach to facilitate intraoperative blood and fluid administration. However, the guide wire was inadvertently lost during catheterization, resulting in a retained intravascular guide wire.

#### Clinical presentation

Despite the retained guide wire, the patient remained asymptomatic with stable vital signs (normal blood pressure, heart rate, and respiratory rate) and an unremarkable chest examination.

#### Diagnostic imaging

**Figure 5:**
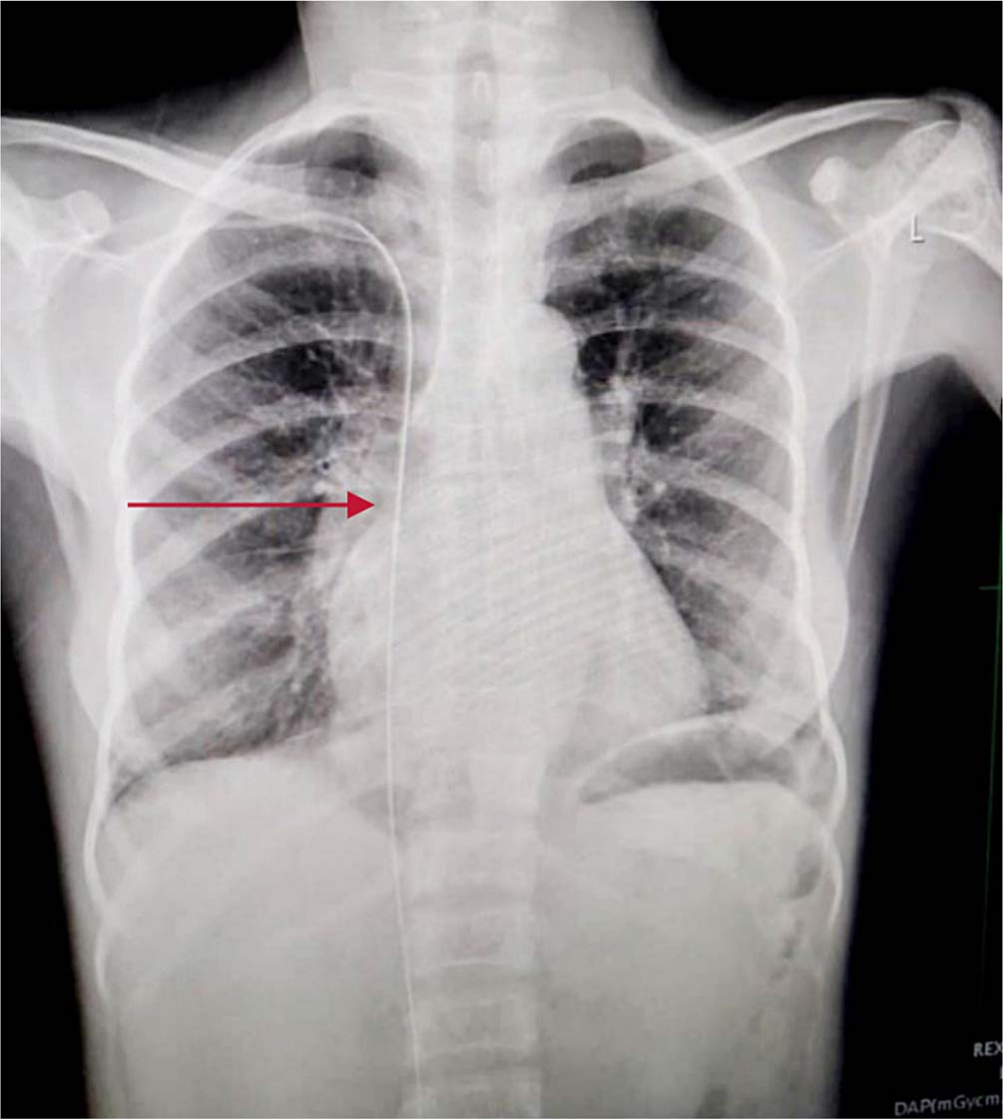
Chest X-ray showing guide wire extending from the right subclavian vein to the SVC and via the right atrium to the IVC.

**Figure 6:**
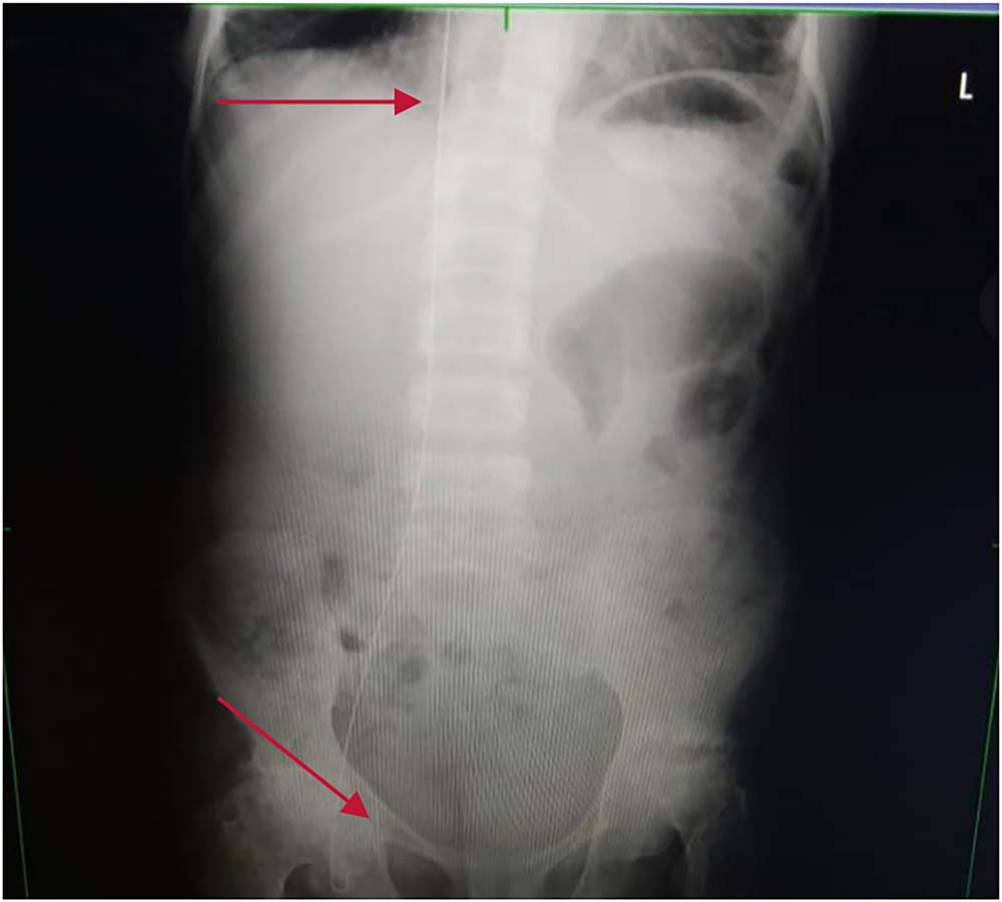
Plain abdominal X-ray extending from the IVC through the right iliac vein to the right femoral vein.

Chest and abdominal X-rays revealed the guide wire extending from the right subclavian vein through the superior vena cava (SVC), right atrium, and inferior vena cava (IVC) to the proximal right femoral vein (Figs 5 and 6).

#### Management and surgical intervention

The patient was counseled and prepared for surgical intervention. Under fluoroscopic guidance, a right groin exploration was performed, revealing the guide wire tip within the right femoral vein just below the inguinal ligament, surrounded by thrombus. A successful guide wire retrieval and thrombectomy were performed via a transverse venotomy after heparinization and vascular control. The venotomy was repaired with prolene 5/0.

#### Post-operative course

The patient had an uneventful post-operative period, with satisfactory recovery and no complications.

## Discussion

Guide wire retention is a rare but clinically significant complication of central venous catheterization. The Seldinger technique, with or without ultrasound guidance, is the most widely accepted method for central venous catheterization. However, the increased availability of interventional ultrasonography in Western countries, which has led to a significant reduction in complications, has not yet been seen in most African countries, where landmark-based methods remain more common.

Since guide wire retention is a completely preventable complication, each case should be treated as an epidemic, necessitating a review of standard operating procedures and learning drills. The index complication occurred due to loss of guide wire control during catheter insertion. Following insertion of the guide wire it is mandatory to hold the guide wire firmly at the skin junction, then, while the catheter is “rail-roaded” through at the distal end, ensure that guide wire control is regained at the distal tip after the full length of the catheter has traversed through the guide wire before releasing the guide wire at the skin junction to insert the catheter. The guide wire must also be inspected after removal to ensure that no fragments were retained. Immediate post-procedure imaging should be recognized as the final step of the CVC placement procedure as it is important to confirm the position of the catheter and detect complications.

Modifications to central venous catheter packaging, such as the CVC plus and WireSafe design can minimize guide wire retention. The CVC plus employed use of bold stamps on the sterile drapes as reminders [4]. The WireSafe features a safety mechanism to prevent guide wire retention and facilitate early identification of complete or fragmented guide wire retention [5].

Extravascular migration of the guide wire is an extremely rare but potentially life-threatening complication of CVC placement. Until the publication of this article, no case of extravascular migration into the thorax has been reported, making the situation particularly challenging. Proper clinical evaluation, stabilization, and prompt imaging are essential for managing a migrated guide wire. Percutaneous retrieval is the preferred removal method, with surgery reserved for removal failure. Fluoroscopic guidance is best practice and may be necessary depending on the location and size of the guide wire. Prevention is key, and adherence to proper techniques and protocols can minimize the risk of guide wire retention and its associated complications.

## Conclusion

This case report emphasizes the need for healthcare providers to be aware of the potential complications associated with CVC placement, including guide wire retention. A multidisciplinary approach to management and prompt recognition of this complication can prevent potentially fatal outcomes.
